# LOWER INCOME IN CAREGIVERS PREDICTS LESS EMOTIONAL WELL-BEING IMPROVEMENT AFTER CAREGIVING ENDS

**DOI:** 10.1093/geroni/igad104.0310

**Published:** 2023-12-21

**Authors:** Julian Scheffer, Claire Yee, Jenna Wells, Yuxuan Chen, Diana Heath, Darius Levan, Jennifer Merrilees, Robert Levenson

**Affiliations:** University of California, Berkeley, Berkeley, California, United States; Mayo Clinic, Scottsdale, Arizona, United States; University of California Berkeley, Berkeley, California, United States; Stanford University, Stanford, California, United States; University of Maryland, College Park, Maryland, United States; University of California Berkeley, Berkeley, California, United States; University of California, San Francisco, San Francisco, California, United States; University of California, Berkeley, Berkeley, California, United States

## Abstract

Lower income in familial caregivers has been shown to associate with increased distress, depression, and burden during active caregiving. However, findings are less clear on whether lower income associates with changes in well-being that occur after caregiving ends. To investigate this question further, we recruited familial caregivers (CGs) of people with neurodegenerative diseases (PWD; N=36). Emotional well-being was assessed using the SF-36 Emotional Well-being and Energy scales both during active caregiving and after caregiving had ended (predominantly due to the PWD’s death or transition to institutional care, ~10 years later). Household income was measured after active caregiving had ended. Overall, CG’s emotional well-being and energy improved between active caregiving and after caregiving had ended (emotional well-being change: t(35) = 2.22, p = .033; energy change: t(35) = 2.88, p = .007). However, this improvement associated with household income, such that when controlling for levels during active caregiving, income associated with worse emotional well-being (B = -6.72, t(33) = -4.56, p < .001) and energy (B = -8.07, t(33) = -4.99, p < .001) in caregivers after caregiving had ended. During the adjustment period after caregiving ends, lower income may limit opportunities for caregivers to take time away from work to mourn the loss of their loved one or address their own health concerns adequately. Thus, low-income caregivers may experience fewer of the benefits associated with these activities. These findings underscore the importance of providing support for lower income caregivers during the critical period after caregiving ends.

